# The microbiota composition drives personalized nutrition: Gut microbes as predictive biomarkers for the success of weight loss diets

**DOI:** 10.3389/fnut.2022.1006747

**Published:** 2022-09-23

**Authors:** Paula Hernández-Calderón, Lara Wiedemann, Alfonso Benítez-Páez

**Affiliations:** Host-Microbe Interactions in Metabolic Health Laboratory, Principe Felipe Research Center (CIPF), Valencia, Spain

**Keywords:** weight loss, dietary interventions, obesity, metabolic disease, gut microbiota, personalized nutrition

## Abstract

The investigation of the human gut microbiome during recent years has permitted us to understand its relevance for human health at a systemic level, making it possible to establish different functional axes (e.g., the gut-brain, gut-liver, and gut-lung axes), which support the organ-like status conferred to this microecological component of our body. The human gut microbiota is extremely variable but modifiable via diet, a fact that allows targeting of microbes through defined dietary strategies to uncover cost-effective therapies to minimize the burden of non-communicable diseases such as pandemic obesity and overweight and its metabolic comorbidities. Nevertheless, randomly controlled dietary interventions regularly exhibit low to moderate degrees of success in weight control, making their implementation difficult in clinical practice. Here, we review the predictive value of the baseline gut microbiota configurations to anticipate the success of dietary interventions aimed at weight loss, mostly based on caloric restriction regimes and oral fiber supplementation. This emergent research concept fits into precision medicine by considering different diet patterns and adopting the best one, based on the individual microbiota composition, to reach significant adiposity reduction and improve metabolic status. We review the results from this fresh perspective of investigation, taking into account studies released very recently. We also discuss some future outlooks in the field and potential pitfalls to overcome with the aim of gaining knowledge in the field and achieving breakthroughs in personalized nutrition.

## Introduction

The human gut microbiota is gaining increasing attention in clinical practice, given the hundreds of reports published in recent years highlighting its contributing role in a wide variety of health states (1, 2). After almost two decades of intensive and rapidly evolving microbiome research, two major traits can undoubtedly be attributed to the gut microbiota. The first one is its profound interindividual influence, and the second one is the enormous influence that diet exerts on it. Regarding the first trait, the great interpersonal variability found in hundreds of human-associated gut microbiota surveys released in the scientific literature makes it difficult to distinguish unique microbial signatures associated with health and disease. Consequently, the so-called “dysbiosis” states presumably associated with several gastrointestinal, mental, and inflammatory maladies–for which causal agents have been examined in the gut microbiota–have an empty and ambiguous meaning (3–5) due to the impossibility of distinguishing concrete microbial communities underlying the disease from the vast variability expected across multiple human populations and settlements.

On the other hand, diet is probably the most important variable–over genetics and other environmental factors (6)–shaping the human intestinal microbiota. Therefore, dietary-based clinical trials constitute a promising scenario to tackle a wide variety of metabolic and inflammatory dysfunctions on the basis of modulating the gut microbiota and thus improving the host-microbe molecular interactions mechanistically demonstrated to modulate human health [e.g., short-chain fatty acids (SCFAs), which have effector roles]. Current investigation paradigms indicate that the Western diet (hypercaloric and enriched in saturated fat and refined sugars) increases opportunistic bacteria, bacterial metabolites (e.g., LPS, TMAO) and inflammatory cytokines while decreasing beneficial bacteria and SCFA production (7). Such a detrimental dietary pattern not only results in an increased risk of chronic diseases (e.g., cardiovascular diseases), but also represents a serious risk for neurodegenerative diseases due to the long-term neuroinflammation favoring the amyloid-β expression and deposition (8, 9). Conversely, plant-based diets, including the Mediterranean diet, are rich in fiber acting as prebiotics stimulating microbial growth in the human gut and are shown to increase SFCAs production and have anti-inflammatory properties, thus decreasing the risk of infectious and non-communicable diseases (7, 10).

Among the multiple macronutrients and dietary compounds and ingredients regularly used in randomized placebo-controlled clinical trials (RCTs) to assess their impact on human health (e.g., unsaturated fat, proteins of plant and animal origin, polyphenols, and whole microorganisms), complex carbohydrates, namely, oligo- and polysaccharides of plant origin, are one of the preferable strategies to modulate the gut microbiota. This preference is based on the lack of genes encoded in the human genome that produce enzymes that break these complex carbohydrates and the contrasting massive molecular circuits for degrading such compounds present in the gut microbiota genomes (11, 12). Such an expanded content of genes encoding carbohydrate-degrading enzymes grants some particular gut microbiota species with outstanding glycolytic versatility (13–15). Moreover, the metabolic products resulting from the microbial fermentation of those complex carbohydrates present in the diet, that is, the SCFAs, have been demonstrated to exert important modulation on host physiology, highlighting this wide variety of dietary fiber compounds as a therapeutic proxy to improve metabolic and immune dysfunctions through the gut microbiota. Nevertheless, the response to dietary interventions with fiber exhibits the same interindividual variability associated with gut microbiota profiling.

On the other hand, calorie-reduced dietary interventions are also repeatedly used in clinical trials to investigate weight loss strategies and to assess to what extent their physiological therapeutic benefits are derived from or translated to the gut microbiome. Caloric restriction diets (CRDs) are known to provide efficacy in reducing adiposity and improving organ function, thereby reducing the risk of non-communicable comorbid diseases (16, 17). Additionally, CRDs protect against host aging and age-related perturbations of the gut microbiota, which are changes that are often associated with pathogen boosting and a proinflammatory status and are therefore detrimental for the progression of several diseases, such as obesity, diabetes, neurological diseases and inflammatory bowel disease (IBD) (18, 19).

Although dietary patterns seem to influence to a larger extent the composition of the gut microbiota and host health by extension, the outcomes have thus far not provided a consensus regarding a particular dietary regimen that is optimal for weight-loss strategies and improving metabolic health. Consequently, the conceptualization of one-diet-fits-all becomes deprecated. Thus, a new perspective of analysis should be adopted with a personalized medicine approach, which can be ascertained via the evaluation of predefined or individualized microbiota profiles in subjects prior to starting dietary regimes aiming for weight loss. Therefore, establishing the links between microbes and dietary ingredients deserves special attention to design such a prospect.

## Links between dietary fiber and gut microbiota

According to the Codex Alimentarius definition, “*dietary fiber consists of carbohydrate polymers with ten or more monomeric units, which are not hydrolyzed by the endogenous enzymes in the small intestine of humans*” (20). Dietary fiber is present in whole grains, fruits, legumes, vegetables, seeds, and nuts. Nevertheless, it is also manufactured [e.g., most oligosaccharides (OSs)] by enzymatic processing of polysaccharides extracted from the food mentioned above or their transformation subproducts. Bacterial utilization preferences for certain oligo- and polysaccharide carbohydrates, their utilization priorities and their respective degradation kinetics not only support species coexistence from an ecological point of view (*Bacteroides thetaiotaomicron* vs. *Akkermansia muciniphila*) (21) but can also inform about the type of complex carbohydrates that can be used in dietary interventions depending on the microbiota present and their abundance. A particular line of investigation is emerging to comprehensively understand the metabolic circuits activated in a species-specific manner by single and complex carbohydrates, and this is of massive relevance to designing personalized medicine strategies based on nutrition.

Fiber is assumed to confer health benefits due to its physicochemical and structural properties (e.g., indigestibility and viscosity) that delay gastric emptying, inhibit nutrient absorption, decrease postprandial glycemic levels, and reduce body cholesterol stores (22). Since it is indigestible by human enzymes, dietary fiber reaches distal portions of the human colon, where it is fermented rapidly by microbes that thrive there to produce different SCFAs. Currently, this biotransformation process is the cornerstone for the design of dietary RCTs aiming to tackle a wide array of maladies affecting the human gastrointestinal tract (GIT), other critical organs, and health as a whole in several ways. The molecular basis underlying the therapeutic transversal approach of dietary fiber is that SCFAs produced in the colon modulate gut barrier integrity, glucose, and lipid metabolism; regulate the immune system, the inflammatory response, and blood pressure; and control enteroendocrine cell networks (23–25).

Conventional RCT design aims to test the effect of particular dietary fiber components and consequently utilizes high purity preparations to isolate their individual effect primarily on clinical parameter indicators of health states (e.g., body weight, blood pressure, glycemia, lipidemia, and motor and cognitive function), and secondary outcomes are frequently used to assess microbiota alterations. In this particular context, manufactured and purified OSs distributed in defined dosages for easy serving preparation are the classic clinical strategy to evaluate diet-based therapies on different diseases with altered gastrointestinal function. The wide diversity of dietary fiber compounds, for which health effects have been tested via RCTs, usually also exhibit an individual-specific response in terms of primary clinical outcomes assessed. Conversely, there is a unique quasi-consensus signal regarding secondary outcomes circumscribed to gut microbiota appraisal. As a general response, *Bifidobacterium* species seem to be predominantly boosted by dietary fiber, with no other consensus signatures captured for alternative species [reviewed in Benítez-Páez et al. (26) and Portuneet al. (27)]. This consistent pattern seen for *Bifidobacterium* species suggests that such beneficial-considered microbes would be the primary gut bacteria degrading the dietary fiber ingested. The above is supported by the fact that *Bifidobacterium* species contain a large number of specialized carbohydrate-active enzymes (CAZy) encoded in their genomes (28). Nevertheless, *Bacteroides* species exhibit the largest glycolytic capability observed in the human gut microbiota (26, 28, 29), which makes their null response in RCTs with dietary fiber perplexing. Hence, the difficulty in discerning other common patterns of gut microbiota modulation through dietary fiber intake could be explained by the Bifidobacteria themselves. Such species increase acetate production after dietary fiber fermentation and can lead to growth stimulation of other bacteria and SCFA production (30, 31). This cross-feeding process could also explain the variation in the gut microbiota response accounting for other multiple species that, in addition to being subjected to Bifidobacteria metabolic-driven interactions, would be strongly conditioned by pre-treatment individual-specific configurations in every RCT.

Although the paradigm of the interactions among the host, diet and microbiota seems to be sound and elegant because of its simplicity and its implications at the systemic level, there is still a great deal of complexity to be unraveled, where the production of the different SCFAs and their relative proportions depend strongly and jointly on the individual composition of the microbiota and the type of dietary fiber administered (32–34).

## Calorie restriction and gut microbiota links

A Western diet is becoming the preferred dietary pattern in Western countries and is widespread in Middle-East and Eastern countries, representing a chronically overfed state and increasing health risk factors and non-communicable chronic diseases (35). These health conditions could lead to metabolic disorders related to the human microbiome, such as an increase in pathogenic gut bacteria, undesirable metabolites and gut permeability, as well as inflammation in the intestine, peripheral organs, and systemically (36). The CRD is one of the commonly used approaches for the treatment of obesity with notable weight loss success (37). The CRD is a nutritional approach that only reduces average daily calorie intake compared to *ad libitum* intake while maintaining acceptable macronutrient proportions (45–65% carbohydrates, 20–35% lipids, and proteins 10–35% in average), sometimes with an additional increase in protein or fiber intake. In general terms, CRD adopted in clinical trials consists in a reduction of 10–30% of caloric intake leaving an average energy intake 1,200–1,800 kcal/day. Globally, the deficit should be of 500–600 kcal/day over baseline caloric intake estimates. Evidence indicates that a CRD may provide a positive impact on gut microbiota diversity (38–40), barrier function, immune and inflammatory responses (41–43), and the production of postbiotic metabolites such as SCFAs (40). Moreover, a CRD has been shown to have an impact on the abundance of all the main phyla of the gut bacteria present in humans, such as *Firmicutes*, *Bacteroidetes*, *Proteobacteria*, *Verrucomicrobia*, and *Actinobacteria* [reviewed in Rinninella et al. (18)]. Nevertheless, as the long-term compliance with a CRD has its limitations, intermittent fasting (IF) or time-restricted feeding regimes consisting of alternating periods of food intake and periods of energy restriction, and limiting feeding to a few hours daily, respectively, have gained increasing attention for implementation during recent years, as they have revealed similar clinical benefits (44–47). Moreover, following a CRD for obese subjects has been shown to have the ability to adjust the gut microbiota composition to those of lean subjects, thus reducing the enormous baseline differences observed (48). Such treatment led to a shift in the gut microbiota composition, increasing the levels of Firmicutes while decreasing most other phyla and consequently increasing the production of SCFAs compared to those of the *ad libitum* controls (49). Clinical trials report that a CRD reduces the Bacteroidetes population in favor of Firmicutes (50). Additionally, in CRD-treated mammals, an overall increase in the relative abundance of probiotic microbes (e.g., *Bifidobacterium* and former *Lactobacillus* species) was detected, which may explain some of the benefits observed in the host (38, 51, 52). On the other hand, some inflammation-inducing microbes could be inhibited with CRD treatment (53), whereas other butyrate-producing microbial strains, such as *Coprobacillus*, *Holdemania*, *Eubacterium cellulosolvens*, and *Clostridium saccharolyticum*, seem to be boosted by CRDs (52).

## The microbiota as a predictive trait for weight loss success in dietary-based strategies

The clinical reports reviewed here and listed in Table 1 were selected as follows: monthly searching in PubMed from October 2021 to July 2022 using the terms “baseline microbiota” OR “pre-treatment microbiota” AND “weight loss.” The retrieved list of publications was manually inspected to assess and explicitly disclose microbiome-based predictive traits for weight loss success of dietary interventions. From the final selected list of publications, their references citing microbiota-based predictive features for weight loss, not recovered from our regular searching, were also incorporated into this review.

**TABLE 1:** Summary of human studies investigating the predictive potential of the baseline microbiota on dietary intervention success.

Dietary pattern	Aim of the study	Study design	Technique	Subjects	Time	Population	Main findings	Reference
CR (deficit of 500 kcal/d)	Studying the role of the microbiome in weight loss and improved hepatic steatosis in response to a CRD	Randomized (R), single-blinded (SB), crossover controlled (CC)	16S rRNA gene sequencing (V4 region)	46	16 weeks	Overweight and obese adults BMI >27 kg/m^2^ 35 men, 11 women Age 20–75 White (19), African American (15), Hispanic (11), Asian (1)	Significant baseline microbiome differences between patients who had at least 5% weight loss compared to the differences in those who did not. Lachnoclostridium was positively associated with hepatic steatosis, and Actinomyces was positively associated with hepatic steatosis and weight.	(66)
CR in the form of the Mediterranean and high-protein diet; crossover intervention	Identifying if different dietary patterns improve metabolic function in a different manner	R, CC	16S rRNA gene sequencing	16	21 days	Insulin-resistant obese (BMI 35–64 kg/m^2^) Only women Age 20–57 Italy	10 microbial genera turned out to be predictive of the difference in glycemic variability between the two diets. *Eubacterium xylanophilum*, Desulfovibrio, Terrisporobacter, *Clostridium* sensu stricto and Coprococcus presented a positive effect on glycemic variability following the HP diet. Ruminococcus, Eggerthella, *Eubacterium hallii*, Lachnoclostridium and Phascolarctobacterium presented a negative association.	(68)
Intermittent calorie restriction (ICR; ∼75% deficit on two non-consecutive days/week) Continuous calorie restriction (CCR; 20% deficit)	Investigating whether ICR or CCR induced alterations in the gut microbiome and to what extent these were associated with overall weight loss irrespective of the dietary intervention	R	16S rRNA gene sequencing (V4 region)	147	50 weeks (12 weeks intervention, 12 weeks maintenance, 26 weeks follow-up)	Overweight and obese adults (BMI ≥25 and <40 kg/m^2^) Age 35–65 50% women Germany	Higher Dorea abundance at baseline negatively correlated with weight loss during intervention.	(62)
CR (∼34% deficit) IF (20% deficit on three non-consecutive days/week)	Examining how clinical measures and the gut microbiota change in response to a weight loss intervention and assessing the cross-sectional and longitudinal relationships between the clinical measures and the gut microbiota.	R, SB	16S rRNA gene sequencing (V3–V4 region)	59	3 months	Overweight and obese adults (BMI 27–45 kg/m^2^) No information given on the sex distribution Age 18–55 USA	The abundance of Subdoligranulum was linearly associated with greater weight loss only among the IF group. The abundances of the Coriobacteriaceae other, Slackia and Eubacterium rectale groups were associated with larger decreases in waist circumference among the IF group. The abundances of Lachnospiraceae other, Holdemanella and Lachnoclostridium were associated with a lower decrease in waist circumference among the IF group.	(64)
CCR vs. IF (both with an energy deficit of 34%) (+physical activity)	Identifying baseline multiomic predictors of weight loss and clinical outcomes within a behavioral-based weight loss trial	R	16S rRNA gene sequencing (V3–V4 region)	56	12 months	Healthy obese or overweight adults (BMI 27–45 kg/m^2^) No information given on the sex distribution Age 18–55 USA	Coprococcus 3 and Ruminococcaceae NK4A214 were advantageous for weight loss. Bacteroides and Lachnospiraceae were disadvantageous for reduced waist circumference. Faecalibacterium and Blautia were associated with greater reductions in triglycerides, while Ruminococcus gnavus was disadvantageous for reductions in TG.	(65)
CR intervention with fiber supplementation (10 g/day inulin + 10 g/day resistant maltodextrin)	Identifying diet-microbiota-host interactions that could account for the metabolic health effects of a dietary intervention	R, DB, PC	Metagenomic shotgun sequencing	80	12 weeks	Overweight and obese adults (BMI 25–40 kg/m^2^) with previous calorie restriction (−500 kcal/day) No information given on the sex distribution Age 18–60 Denmark	Baseline abundances of *Bacteroides fragilis* or *Bacteroides ovatus* (ef_mOTU_v2_1073) were negatively related to the weight loss during the CRD.	(40)
CR with high-protein diet in the form of formula (810 kcal/day; 44% protein)	Investigating how the gut microbiota change during a total meal replacement low-energy diet (LED) and determining their associations with host response	R	16S rRNA gene sequencing (V3–V4 region)	211	8 weeks	Overweight adults (BMI >25 kg/m^2^) with prediabetes 55 men, 156 women Age 25–70 Caucasian (194), Polynesian (13), Asian (3), others (1)	The higher relative abundances of Clostridium sensu stricto 1, Ruminococcaceae UCG-003 and Parabacteroides at baseline positively correlated with fat loss, and Erysipelotrichaceae UCG-003 was negatively correlated.	(67)
Low-carbohydrate diet (LCD)	Verifying the hypothesis that the gut microbiota contributes to the inconsistent outcome under an LCD	R	Metagenomic shotgun sequencing	51	12 weeks	Overweight (BMI 24–28 kg/m^2^) and obese (BMI >28 kg/m^2^) adults No information given on the sex distribution Age 21–59 China	The high relative abundance of Bacteroidaceae, especially Bacteroides, at baseline was positively correlated with weight loss efficacy.	(57)
LCD vs. LFD (limiting either carbohydrates or fat to ∼20 g/d)	Determining if the baseline microbiota composition/diversity is associated with weight-loss success	R	16S rRNA gene sequencing	49	12 months	Healthy overweight or obese (BMI 28–40 kg/m^2^) adults No information given on the sex distribution	The baseline microbiota composition was not predictive of weight loss.	(79)
High-fiber (30 g/day) supplemented diet	The researchers hypothesized that (I) subjects with a higher Prevotella/Bacteroides ratio would improve body weight control on the AXOS supplemented diet compared to the PUFA-enriched diet and that (II) some species with AXOS-degrading capacity would specifically predict body weight changes	R, CC	Metagenomic shotgun sequencing	29	4 weeks	Overweight and obese adults (BMI 25–40 kg/m^2^) No information given on the sex distribution Age 18–60 Denmark	Subjects who controlled weight tended to have a lower abundance of *Bacteroides cellulosilyticus* when consuming AXOS.	(56)
Low-resistant starch intervention: 9.2 ± 1.1 g of resistant starch High-resistant starch intervention: 3.7 ± 3.0 g of resistant starch	Assessing baseline characteristics to predict the postprandial glucose response (PPGR) in individuals following an intervention of low- and high-resistant starch potatoes and developing a precision nutrition model to predict the PPGR in overweight women	R	16S rRNA gene sequencing (V3–V4 region)	30	–	Overweight women (BMI 25–40 kg/m^2^) No information given on the sex distribution Age 18–40 USA	Relative abundance of Faecalibacterium is negatively associated with glucose iAUC in both.	(69)
Hypercaloric diet (excess of 1,000 kcal/day) rich in saturated fat, unsaturated fat or simple sugars	Studying (I) the effect of short-term overfeeding on the human gut microbiota in relation to baseline and overfeeding-induced liver steatosis and (II) whether the baseline microbiota composition is associated with the overfeeding-induced increase in liver fat	R	16S rRNA gene sequencing (V3–V4 region)	38	3 weeks	Overweight and obese adults (BMI 25–40 kg/m^2^) 17 men, 21 women Age 48 ± 2 years Finland	Baseline prevalence and mean abundance of Desulfovibrionacea, especially the genus Bilophila, were significantly higher in subjects with an overfeeding-induced increase in the liver fat.	(71)

As summarized in the previous sections, caloric restriction and fiber-based orally administered ingredients are the preferred weight-loss strategies in RCTs and seem to exert profound effects on gut microbiota configurations. However, the prominent feature of such controlled interventions is that no consensus outcomes are reached, and an elevated variability in weight reduction is seen among participants. Consequently, these results are prominently linked with gut microbiota profiling, given the strong influence of diet on intestinal microbiota structure.

**FIGURE 1:**
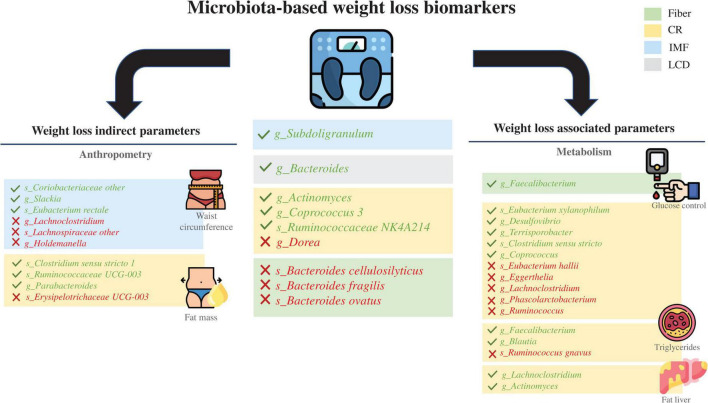
Schematic results of all the clinical trials reviewed. Taxa in green indicate a positive predictive manner, and taxa in red indicate a negative predictive manner. Dietary interventions and their influence on obesity-related metabolic parameters with a focus on weight loss as well as the correlated gut microbiota taxa at the genus or species level. CR, caloric restriction; IF, intermittent fasting; LCD, low-carbohydrate diet.

Accumulating evidence regarding the interaction between the microbiota and weight-loss interventions in overweight (generally classified as BMI >25 kg/m^2^) or obese (BMI 28–30 kg/m^2^) adults reveals that differences in the intrinsic baseline gut microbial profile could likely play a conditioning role in the success of health interventions (54). In this regard, a novel concept in microbiome research that has emerged in the last 4 years includes considering the baseline microbiota as a predictor of weight loss success following a low-calorie diet in RCTs. However, some studies have looked at other perspectives, such as IF or fiber supplementation. The characteristics of the collected studies and their specific results are listed in Table 1, and an outline of the primary outcomes with the related taxa is presented in Figure 1.

*Bacteroides* and *Prevotella* species are the most studied microbial genera, probably because they are the dominant stable clusters of bacterial communities in the human gut. Researchers from the University of Copenhagen were pioneers who inferred weight-loss success in RCTs using such features and have focused their research on personalized dietary strategies with an “enterotype” perspective, emphasizing the importance of the *Prevotella*/*Bacteroides* balance (54). From the information gathered from genomic analyses and targeted *in vitro* studies, *Prevotella* species seem to favor fiber degradation over their *Bacteroides* counterparts, and they are associated with better weight modulation in response to increased dietary fiber intake. As a proof of concept, the *Prevotella* abundance at baseline has been shown to be a positive predictive biomarker for the success of weight loss (55). In contrast, a high abundance of *Bacteroides cellulosilyticus* has been found to be a potential predictor for failure of weight-loss intervention following oral administration of arabinoxylan-oligosaccharides (AXOS) (56). Similarly, the baseline abundance of *Bacteroides fragilis* or *Bacteroides ovatus* was negatively related to weight loss after a calorie restriction intervention with fiber supplementation in a previous multiomics study completed by our group (40). Nevertheless, other authors have described *Bacteroides* species as a positive predictor of individual weight loss after a short-term low-carbohydrate dietary intervention (57). In accordance with this, *Bacteroides dorei* (together with *Blautia wexlerae*) was found to be a strong predictor for weight loss when present in higher relative abundance at baseline before following a high-carbohydrate and high-protein CRD (58). These contrasting results regarding the *Bacteroides* genus may be due to differences in methodology, study design and population characteristics, but it is also necessary to obtain a more detailed microbiota profiling outlook at the species level.

Bearing this in mind, to obtain more precise insight into how the baseline microbiota can be predictive, we focused on studies aimed at the evidence at the species level. Nonetheless, research that tackles that perspective is currently insufficient, and there are considerable heterogeneities in study design, population and methodologies. Furthermore, current methods are not sufficiently discriminatory to discern between closely related species, and the genetic and metabolic variability revealed in recent years indicates that identification at the subspecies level could also be a critical aspect to take into account for predictive aims (e.g., *Prevotella copri* clades, *Eubacterium hallii* clusters and *A. muciniphila* subspecies) (59–61) and should offer a more intricate perspective on predictions aimed at designing personalized medicine strategies.

Beyond the *Prevotella/Bacteroides* ratio, a low abundance of the *Dorea* genus at baseline has been shown to be predictive of subsequent weight loss following intermittent calorie restriction, nullifying the predictive capability of the *Prevotella/Bacteroides* ratio or gut microbiota richness in this particular nutritional context (62). The predictive value of the *Dorea* species abundance (*Dorea longicatena*) was also supported, but in different directions, by its positive correlation with the BMI loss ratio after CRD intervention (63). This type of disparity in results is quite frequent in microbiota assessment under particular nutritional regimes and could be the result of the technical limitations when defining species-level taxonomy associations. In addition, when CRD and IF interventions are compared, they also produce discrepancies in the predictive value of certain taxonomy features. For instance, the abundance of *Subdoligranulum* species was associated with more significant weight loss only after IF intervention (64). A short-term weight loss intervention based on a CRD reducing caloric intake by 34% showed improved weight reduction linked to *Coprococcus* species baseline abundance, while *Bacteroides* and Lachnospiraceae species were negatively related to waist circumference reduction (65).

From a different perspective, Dong and coworkers reported significantly different outcomes related to the variability between patients with at least 5% weight loss on a calorie-restricted diet compared to those with less response. The former group had less *Escherichia/Shigella*, *Klebsiella*, *Megasphaera*, *Sellimonas*, and *Lactobacillus* and more *Collinsella* as well as an unidentified genus from the Christensenellaceae family compared to those that did not respond as well to a calorie-restricted diet. In addition, *Actinomyces* was shown to be a negative predictor for weight loss following the given intervention, and it seemed to be predictive of the development of hepatic steatosis (66).

## Alternative diet-based strategies showing the predictive value of baseline microbiota features

A recently published study did not find baseline microbiota to be a good predictor of weight loss, but it showed that the baseline microbiota was a good predictor of indirect measures of weight loss, such as fat mass lost, after a high-calorie restriction regimen with a high-protein diet. Jian et al. (67) observed that high relative abundances of *Clostridium sensu stricto*, Erysipelotrichaceae and *Parabacteroides* species were negatively correlated with fat mass loss. Similarly, other authors have focused more on different outcomes that frequently accompany weight loss as the metabolic parameters improve. However, those clinical outcomes of interest also seem predictable using baseline gut microbiota and diet configurations, which allows us to broaden the possibilities of where the basal microbiota could play an important role as a biomarker. For instance, Tettamanzi et al. detected at least ten microbial species, including *Eubacterium xylanophilum*, *Clostridium* sensu stricto, *E. hallii*, and *Lachnoclostridium*, suggesting that they play an important role in host glucose homeostasis. *Coprococcus*, for example, might exert a positive effect through the production of SCFAs. Moreover, *Lachnoclostridium* species might metabolize precursors of trimethylamine and its oxide, which negatively regulate glucose metabolism and insulin sensitivity (68). Another study assessed baseline characteristics to predict the postprandial glucose response and revealed that the relative abundance of *Faecalibacterium* is negatively associated with plasma glucose after the consumption of high- and low-resistant starch (69). This finding could be of special interest since glucose metabolism impairment is one of the most common and problematic issues in overweight and obese patients.

The well-recognized beneficial human gut microbe *A. muciniphila* has also been identified as a diagnostic or prognostic tool in the context of predicting dietary intervention success. Consequently, *A. muciniphila* abundance at baseline showed correlations with improvements in body fat distribution, fasting plasma glucose, plasma triglycerides and insulin sensitivity after a CRD (70).

Nevertheless, not all studies are directed toward clarifying the relationship between the baseline gut microbiota and CRD or weight loss strategies. Strikingly, a randomized crossover trial published in 2021 aimed to study the effects of short-term overfeeding on the human gut microbiota and liver steatosis and showed that fat accumulation in the liver is linked to a higher baseline abundance of *Bilophila* species (71).

## Discussion

Current research indicates that the baseline microbiota may act as a predictor of weight loss, which could have implications for clinical practice such that we could use the microbiota as a biomarker for predicting successful clinical interventions in the future. In summary, we have observed that elucidation of predictive baseline microbiota traits for weight loss dietary strategies is variable, and no consensus signals can be intuited. Undoubtedly, such an observation likely results from the recognized inter-individual variability of gut microbiota profiles and the variability of weight loss diets explored in this review. The literature reviewed allows us to hypothesize that we can target the microbiota with some dietary interventions using high-fiber or calorie restriction regimes to modulate the microbiota and improve the outcomes of proposed cost-effective therapies for weight loss. However, the association between the predictive traits of the baseline gut microbiota and the success of dietary interventions requires more research. In this regard, we have recently described that a sex-based perspective should be adopted in all RCTs to improve the precision of predictions. We have found, from a multiomic perspective, that women and men responded disparately to a CRD regimen with fiber supplementation (72). Furthermore, the recent work published by Cuevas-Sierra and coworkers analyzed the baseline microbiota predictive value for weight loss dietary strategies upon low-fat and high-protein regimes separately. When sex was included in the data modeling, the observed predictions were notably improved (73). The above results are strongly suggestive of directions to adopt in future studies. The recognized influence of specific covariates, directly or indirectly linked to dietary patterns, stresses the importance of considering factors such as sex, age and nutritional habits to improve biomarker predictions. Altogether, integration of such covariates into the predictive models will permit the creation of more robust associations and defining discrete diets for individuals depending on their gut microbiota configuration to reach weight loss; thus we will move toward abandoning of the one-diet-fits-all concept, as well as the delineation of personalized nutrition in the context of precision medicine to improve weight loss strategies and reduce the burden of non-communicable disease associated with obesity and overweight in human populations.

## Future perspectives

We want to express our conviction that there is an evident need to improve microbiota profiling in clinical and preclinical studies toward describing taxonomy inventories at the species level and minimizing potential issues during biocomputational processing of microbiome data. The level of technical and methodological information found in most microbiome studies is insufficient and does not contribute positively to solving contrasting and disparate results frequently found in the literature. Important initiatives have emerged to tackle such pitfalls, including the introduction of the STORMS Checklist as a guideline for microbiota assessments in clinical studies (74). However, beyond the issues the above-claimed standard will solve, there is an underlying technical limitation to microbiota profiling assessments, which makes it unsuitable to reach the level of detail currently required and for the advances in this research. For example, data generated via the short-read sequencing of 16S rRNA gene amplicons, widely used in microbiome surveys, cannot reach a remarkable proportion of annotations at the species level. The only technology able to provide high-quality information in this regard is shotgun sequencing, which is more expensive and makes studies with a large set of samples unfeasible. Consequently, steps forward are needed to establish a middle-ground approach that will permit taxonomy resolution with lower costs. In this regard, our group has proposed a cost-effective strain-level assessment using *rrn* long-amplicons as a promising technique, which could help to solve the issues stated above (72, 75). Moreover, a very comprehensive repository is also available to complete this advanced method of analysis (76).

With the proper control of covariation in microbiota data using host-associated variables and improvements in the annotation of species, we should advance the knowledge and better explain the variability in the baseline characteristics predictive of success of different strategies. Although such variability could correspond and be representative of the well-known individual characteristics of the microbiota configurations, the metabolic landscape set and guided by those predictive traits cannot be disregarded since functional redundancy is also characteristic of gut microbiomes (77). With the resolution, at the species or strain level, of predictive microbes that permit us to anticipate the result of a diet-based weight-loss strategy, we could start to define carbohydrate (simple or complex) preferences in such species to disclose the metabolic landscapes in different nutritional environments guiding adiposity reduction. Consequently, future studies to define complex carbohydrate utilization preferences by gut microbes (15, 78), evaluate different effector molecules produced by gut microbes (33, 34), and assess the production of SCFAs depending on fiber ingredients and the physiological environment (32) will all be necessary to integrate such knowledge into clinical practice.

## Author contributions

AB-P conceived and coordinated this mini-review. All authors compiled and critically analyzed the clinical trial studies reviewed in this work.
